# Prevalence of adhesions and associated postoperative complications after cesarean section in Ghana: a prospective cohort study

**DOI:** 10.1186/s12978-017-0388-0

**Published:** 2017-11-02

**Authors:** Mercy A. Nuamah, Joyce L. Browne, Alexander V. Öry, Nelson Damale, Kerstin Klipstein-Grobusch, Marcus J. Rijken

**Affiliations:** 1 Department of Obstetrics and Gynecology, School of Medicine and Dentistry, College of Health Sciences, University of Ghana, P.O. Box GP 4236, Accra, Ghana; 2 University Medical Center Utrecht, Utrecht, The Netherlands; 3 Department of Obstetrics and Gynecology, Korle-Bu Teaching Hospital, Accra, Ghana; 4 Division of Epidemiology & Biostatistics, School of Public Health, Faculty of Health Sciences, University of the Witwatersrand, Johannesburg, South Africa; 5 Department of Obstetrics and Gynecology, Division of Woman and Baby, University Medical Center Utrecht, Utrecht, The Netherlands

**Keywords:** Cesarean sections, Adhesions, Complications, Low- and middle-income countries

## Abstract

**Background:**

The global increase in Cesarean section rate is associated with short- and long-term complications, including adhesions with potential serious maternal and fetal consequences. This study investigated the prevalence of adhesions and association between adhesions and postoperative complications in a tertiary referral hospital in Accra, Ghana.

**Methods:**

In this prospective cohort study, 335 women scheduled for cesarean section at Korle-Bu Teaching Hospital in Accra, Ghana were included from June to December 2015. Presence or absence of adhesions was recorded and the severity of the adhesions was scored using a classification system. Associations between presence and severity of adhesions, postoperative complications, and maternal and infant outcomes at discharge and 6 weeks postpartum were assessed using multivariate logistic and linear regression analysis.

**Results:**

Of the participating women, 128 (38%) had adhesions and 207 (62%) did not. Prevalence of adhesions increased with history of caesarean section; 2.8% with no CS but may have had an abdominal surgery, 51% with one previous CS, 62% with >1 CS). Adhesions significantly increased operation time (mean 39.2 (±15.1) minutes, absolute adjusted difference with presence of adhesions 9.6 min, 95%CI 6.4-12.8), infant delivery time (mean 5.4 (±4.8) minutes, adjusted difference 2.4 min, 95%CI 1.3-3.4), and blood loss for women with severe adhesions (mean blood loss 418.8 ml (±140.6), adjusted difference 57.6 ml (95%CI 12.1-103.0). No differences for other outcomes were observed.

**Conclusion:**

With cesarean section rates rising globally, intra-abdominal adhesions occur more frequently. Risks of adhesions and associated complications should be considered in counseling patients for cesarean section.

**Electronic supplementary material:**

The online version of this article (10.1186/s12978-017-0388-0) contains supplementary material, which is available to authorized users.

## Plain English summary

Cesarean sections (CS) are potentially life saving for mother and child. The percentage of babies born through CS is rising globally, including in low- and middle-income countries. As the procedure is not without risks or complications, it is important to understand how often these arise. Adhesion (scar tissue) formation can happen after any abdominal surgery and occurs often after a CS. A major problem is that adhesions make (future) CS more difficult.

What did we study? We observed CS of 335 women in in Korle-Bu Teaching Hospital (KBTH), a large referral hospital in Accra, Ghana. We recorded whether adhesions were present, how severe they were, and whether they were associated with risks for mother or baby.

What did we find out? Over a third of women (38%) had adhesions, and this occurred more often if they had had a previous CS or abdominal surgery: about two-third of women with more than one CS had adhesions. The surgeries of women who had adhesions were on average longer (by 9.6 min), the time until the baby was born was longer (2.4 min), and women with severe adhesions had more blood loss (57 ml). No differences for other outcomes for mother or baby were observed.

What do we conclude from these findings? A CS can result in post-operative complications such as adhesions. These have implications for future pregnancies: adhesions result in longer duration of repeat CS and more blood loss. Given the rise in CS and their associated complications, it is important to avoid unnecessary CS.

## Background

Cesarean section (CS) is the most frequently performed operation worldwide and the rates of CS delivery have been rising rapidly over the past few decades [1–3]. Currently, the long standing World Health Organization (WHO) advice of 10-15% of deliveries by CS [4] or the recently suggested optimum of 19% [5] is exceeded in many high-income (average rate of 27%) and low to middle income settings (between 3% to 29%) [3, 6–8]. This increase has been attributed to improved safety of the procedure, fewer vaginal births after cesarean (VBAC), preferred method of delivery in breech presentation or suspected cephalopelvic disproportion, more high-risk pregnancies, an increase in CS performed at maternal request, the medicolegal environment of obstetrics, and changes in practice patterns of providers [9–11].

The prevalence of CS-associated short- and long-term complications increases with each additional CS and these include postoperative bleeding and infection, abdominal adhesions, placenta accreta and surgical injury [9, 12, 13]. In addition, future pregnancies may be complicated by uterine scar rupture with potential serious maternal and fetal consequences [14].

Adhesions are reported as a frequent complication of CS [12], and can result in abdominal discomfort, pain and associated lower quality of life [15]. In the long term, adhesions may complicate future CS because of increased difficulty of the surgical procedure resulting in complications such as bladder damage and prolonged duration of surgery [16]. Especially with an emergency (repeat) CS, these often unexpected difficulties can result in adverse perinatal and maternal outcomes such as birth asphyxia and maternal exhaustion. Despite the increases in (repeat) CS, few studies exist on the prevalence of adhesions and associated maternal and perinatal outcomes, especially in Sub-Saharan African settings.

## Methods

### Aims

This study aimed to investigate the prevalence of adhesions in women with and without previous CS, and the association between adhesions and maternal and neonatal outcomes 3 days and 6 weeks postpartum.

### Study design and setting

This prospective cohort study was conducted between June and December 2015 at the Korle Bu Teaching Hospital (KBTH); a tertiary referral hospital in Accra, Ghana. KBTH has between 10.500-11.000 deliveries annually and a CS rate of 43% in 2013 (unpublished data, annual statistics report KBTH 2013), more than Ghana’s average of 4% in 2003 [2].

### Participants

All women >18 years of age admitted for elective or emergency CS were eligible candidates. Recruitment primarily took place on weekdays from 9:00 to 17:00, based on labor ward coverage by research assistants. All participants were followed until discharge from the hospital, usually 3 days postpartum. A subset of women and infants recruited during the first 5 weeks of the study were evaluated at 6 weeks during their routine postnatal appointment. Reporting was according to STROBE guidelines [17].

### Sample size calculation

The sample size was calculated based on an estimated proportion of women undergoing a repeat CS of 30% and the assumption that adhesions were present in most of the repeat CS women, but not primary CS women [12].

To detect a significant difference (*p* < 0.05) in adhesion prevalence between women with primary versus repeat CS with a z-score of 1.96 and a power of 95% (2-tailed alpha = 0.05), 322 participants were required [18]. To account for a 10% contingency, 350 women were to be included.

### Variables

Trained research assistants collected data using standardized questionnaires. Pre-operatively, demographic and socio-economic data (age, marital status, education level) was collected. Medical and obstetric history was obtained from the patients’ maternity booklets, labor ward records and doctors notes and included the following: gestational age at delivery (based on ultrasound gestational age determination in first trimester or at booking, known medical conditions including presence of fibroids, use of medication (and type), mid-pregnancy weight, previous hospital admittances and/or surgeries, number of previous pregnancies and deliveries including abortions and miscarriages, any problems during previous pregnancies or deliveries as well as during the current pregnancy, indication for and number of previous cesarean sections and the duration and location of hospital stay after the most recent CS. Indication for CS was classified as maternal, combined maternal/neonatal or neonatal. Maternal reasons included: antepartum hemorrhage, large fibroid in situ obstructing vaginal delivery, previous CS, (pre)-eclampsia, complicated obstetric history (e.g. perinatal death), maternal wish and gestational diabetes (ketoacidosis). Combined maternal/neonatal included: cephalopelvic disproportion (CPD), placenta previa, unsuccessful VBAC, and estimated birth weight > 4000 g. Neonatal indications were breech presentation, fetal distress, twin pregnancy, premature rupture of membranes (PROM), and severe oligohydramnion.

#### Exposure variable

Presence of adhesions was established during CS by the operating gynecologist or resident, and assessed by two research assistants trained in adhesion scoring (Table 1) using the adhesion classification scheme developed by Tulandi & Lyell [17]. In this scheme, adhesion is graded by location, consistency and size. Filmy (transparent and easily dehisced) adhesions larger than 6 cm wide were allotted maximum of 4 points whereas dense (difficult to separate) adhesions were allotted minimum of 4(<3 cm) and maximum of 16(>6 cm) points if they occurred between the uterus and bladder, abdominal facial or omentum. Irrespective of size, a filmy adhesion between omentum and abdominal facial was allotted 2 points, and a dense adhesion 8 points. With respect to other pelvic structures that interfere with delivery, filmy and dense adhesions were graded with 4 and 8 points, respectively. Severity of adhesion was dichotomized as absent or present, and classified as “mild” or “severe” based on adhesion scores below and above the median (16 points, range 0-64 points).

**Table 1:** Adhesion classification system according to Tulandi & Lyell [17]

Adhesions	Consistency of adhesions	<3 cm	3-6 cm	>6 cm
Between uterus and bladder	Filmy	1	2	4
	Dense	4	8	16
Between uterus and abdominal fascia	Filmy	1	2	4
	Dense	4	8	16
Between uterus and omentum	Filmy	1	2	4
	Dense	4	8	16
Between omentum and abdominal fascia	Filmy		2	
	Dense		8	
Adhesions to other pelvic structures that interfere with the delivery	Filmy		4	
	Dense		8	

### Outcome variables

Perioperative outcomes obtained were operation time (measured from skin incision to skin closure), infant delivery time (incision until time of birth), perioperative blood loss, Apgar scores at 5 min (<7 or ≥7), and need for neonatal intensive care unit (NICU) admission.

Postpartum outcomes until discharge collected were the length of the hospital stay and occurrence of wound infection. Information during their 6 weeks postpartum follow up visit collected was: occurrence of fever or wound infection, need for (continued) use of painkillers, abnormal bleeding (persistent spotting or bleeding clots as determined by attending clinician), ability to resume daily activities, and the need for a healthcare professional consultation in the 6 weeks postpartum for mother and /or her infant.

### Statistical analysis

Baseline characteristics were presented for all participants with means and standard deviations for continuous variables, and frequency and percentage for categorical variables. To compare women with and without adhesions, baseline characteristics differences were assessed using Student’s T-tests, Fisher’s exact tests, Pearson’s Chi square or one-way ANOVA, where appropriate.

The association between adhesions and postoperative and postpartum outcomes was assessed using logistic and linear regression analyses. Continuous outcomes were presented as absolute differences with 95% confidence interval (95% CI), dichotomous outcomes as Odds ratios (ORs) with 95% CI.

Two confounders were selected for inclusion in the multivariate regression analysis based on known associations with the determinants and outcomes: weight at 20 weeks gestation and presence of uterine fibroids. Values for confounders with >15% incomplete data were imputed using multiple imputations. Missing outcome data were considered lost at random and excluded for analysis.

Post-hoc analyses to explore the relationship between adhesion severity and outcomes were performed in a similar regression approach, but with adhesions as a continuous score and by the classifications of mild and severe.

The level of statistical significance determined at *p* < 0.05. All statistical analyses were performed with STATA (Version 11, *StataCorp*, College Station, Texas).

## Results

**Fig. 1:**
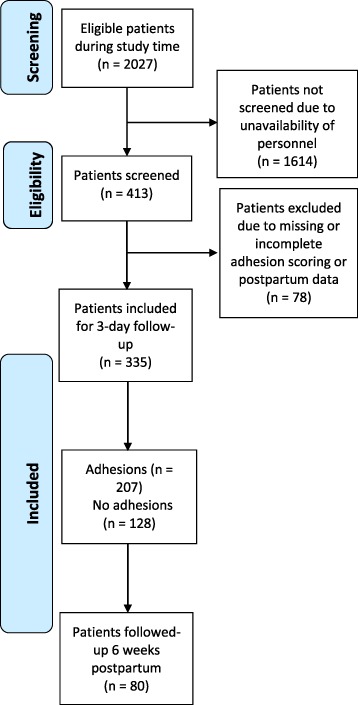
Flow diagram of participant inclusion

Between June and December 2015, 413 participants were included in the study, 20.4% of the 2027 cesarean deliveries performed at Korle Bu Teaching Hospital in this period. Seventy-eight patients were excluded due to missing or incomplete data on adhesion score or postpartum outcomes, resulting in a total of 335 women included for analysis (81.1%) (Fig. 1). All included patients were followed-up until discharge, and 80 women (23.8%) were followed up 6 weeks postpartum.

### Prevalence and severity of adhesions

**Fig. 2:**
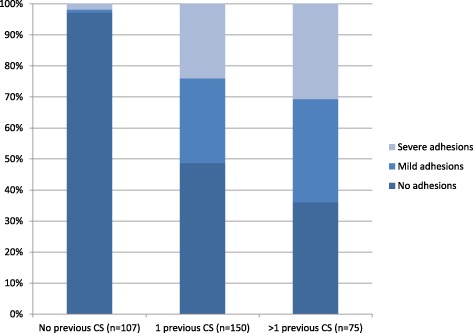
Presence and severity of adhesions by number of previous cesarean section

Of the 335 included women, 207 (62%) did not have adhesions and 128 (38%) did. Prevalence of adhesions increased with history of caesarean section (0% with no history of CS or abdominal surgery, 2.8% (3/107) with no history of CS but may have had a previous abdominal surgery, 51% (77/150) of women who had one previous CS, and 62% (48/78) of those with two or more CS (Fig. 2, Additional file 1: Table S1).

In Table 2 the baseline socio-demographic, obstetric and health characteristics by presence or absence of adhesions (Additional file 2: Table S2 by adhesion category) are presented. Women with adhesions were significantly older (32.3 ± 4.3 vs. 30.6 ± 5.2 years) and less likely to live in the Greater Accra region (107/125, 85.6% vs.191/206, 92.7%). Women with adhesions more often were multiparous (125/127, 98.4% vs.144/207, 69.6%), and had a previous CS (125/128, 97.7% vs.103/207, 49.8%). Seventeen women had a previous abdominal surgery other than CS, all in the adhesions group (17/128, 13.3%) Women with adhesions were more likely to currently have an elective CS (59/128, 46.1% vs. 68/207, 32.9%) on maternal indication (89/126, 70.6% vs. 84/203, 41.3%). Women did not differ in education, marital status, mid-pregnancy weight, present of uterine fibroids or indication of current CS. The prevalence and severity of adhesions increased with each additional CS (Fig. 2).

**Table 2:** Baseline characteristics of participating women

	All, *n* = 335	No adhesions, *n* = 207	Adhesions, *n* = 128	*P*-value
Socio-demographic characteristics				
Age (years)	31.2 ± 4.9	30.6 ± 5.2	32.3 ± 4.3	<0.01
Current living in Greater Accra region (n, %)	298 (90.0)	191 (92.7)	107 (85.6)	<0.01
Married, engaged or living together (n, %)	286 (87.2)	174 (87.0)	112 (87.5)	0.90
Education level (n, %)				0.99
No education	28 (8.5)	17 (8.4)	11 (8.6)	
Primary school	94 (28.5)	59 (29.1)	35 (27.6)	
Secondary school	113 (34.2)	69 (34.0)	44 (34.7)	
Tertiary school	95 (28.8)	58 (28.6)	37 (29.1)	
Obstetric and medical history				
Parity (n, %)				<0.01
0	65 (19.5)	63 (30.4)	2 (1.6)	
1-4	259 (77.5)	137 (66.2)	122 (96.1)	
5-9	10 (3.0)	7 (3.4)	3 (2.4)	
Number of previous CS (n, %)				<0.01
0	107 (31.9)	104 (50.2)	3 (2.3)	
1	150 (44.8)	73 (35.3)	77 (60.2)	
2	61 (18.2)	27 (13.0)	34 (26.6)	
3	17 (5.1)	3 (1.5)	14 (10.9)	
Indication for previous CS (n, %)				0.07
Maternal	61 (28.9)	21 (21.4)	40 (35.4)	
Maternal-neonatal	82 (38.9)	40 (40.8)	42 (37.2)	
Neonatal	68 (32.2)	37 (37.8)	31 (27.4)	
Previous surgery (n, %)	17 (5.1)	0 (0)	17 (13.3)	<0.01
Uterine fibroids	12 (3.6)	6 (2.9)	6 (4.7)	0.39
Mid-pregnancy weight in kg (mean, SD)	74.2 (14.5)	73.8 (15.0)	75.0 (13.3)	0.49
Current delivery				
Gestational age at delivery in weeks (n, %)^⥾^				0.62
≤ 37	78 (25.4)	51 (27.1)	27 (22.7)	
38 – 41	220 (71.7)	130 (69.7)	89 (74.8)	
≥ 42	9 (2.9)	6 (3.2)	3 (2.5)	
Type of current CS				0.02
Elective	127 (37.9)	68 (32.9)	59 (46.1)	
Emergency	208 (62.1)	139 (67.2)	69 (53.9)	
Indication for current CS (n, %)				<0.01
Maternal	173 (52.9)	84 (41.8)	89 (70.6)	
Maternal-neonatal	83 (25.4)	57 (28.4)	26 (20.6)	
Neonatal	71 (21.7)	60 (29.9)	11 (8.7)	

*CS* cesarean section, *n* number, *SD* standard deviation

### Association between adhesions and complications

Presence of adhesions significantly increased the operation time (mean duration 39.2 (±15.1) minutes, absolute adjusted difference with presence of adhesions 9.6 min (95% CI 6.4-12.8) and 14.2 min (95% CI 10.1-18.3) for severe adhesions). Infant delivery time was on average 5.4 (±4.8) minutes, the adjusted difference 2.4 min (95% CI 1.3-3.4) with presence of adhesions and 3.1 min (95% CI 1.7-4.4) for severe adhesions) compared to no adhesions (Tables 3 and 4, Additional file 3: Table S3). Blood loss was not-significantly higher for women with adhesions, but for women with severe adhesions there was significantly more peri-operative blood loss recorded (mean blood loss 418.8 (±140.6) ml, adjusted difference 57.6 ml (12.1-103.0) for severe adhesions). No differences for other perioperative or postpartum maternal and neonatal outcomes were observed (Tables 3 and 4).

**Table 3:** Maternal and neonatal outcomes in women, by adhesion status

	All	No adhesions	Adhesions	*P* – value
	*n* = 335	*n* = 207	*n* = 128	
Maternal outcomes				
Perioperative outcomes (*n* = 335)				
Operation time (minutes), mean, SD	39.2 (15.1)	35.4 (13.7)	45.3 (15.2)	<0.01
Infant delivery time (minutes), mean, SD	5.4 (4.8)	4.5 (4.5)	6.9 (4.8)	<0.01
Perioperative blood loss (ml), mean, SD	418.8 (140.6)	408.5 (167.6)	434.8(140.5)	0.14
Postoperative outcomes at time of discharge(*n* = 335)				
Length of hospital stay (days), mean, SD	3.8 (1.0)	3.9 (0.9)	3.7 (1.1)	0.17
Wound infection (n, %)	4 (1.2)	4 (2.0)	0 (0.0)	0.30
6 weeks postpartum (*n* = 80)				
Fever (n, %)	4 (5.0)	2 (4.7)	2 (5.4)	1.00
Wound infection (n, %)	8 (7.6)	4 (6.6)	4 (8.9)	0.72
Use of painkillers (n, %)	3 (3.7)	1 (2.3)	2 (5.4)	0.59
Resume daily activities (n, %)	56 (70.0)	31 (72.1)	25 (67.6)	0.66
Abnormal bleeding (n, %)	10 (12.5)	5 (11.6)	5 (13.5)	0.80
Need for healthcare professional (n, %)	12 (15.0)	8 (18.6)	4 (10.8)	0.37
Neonatal outcomes				
Perioperative outcomes (n = 335)				
Apgar score < 7 at 5 min. (n, %)	20 (6.0)	13 (6.3)	7 (5.5)	0.75
NICU admissions (n, %)	42 (13.4)	28 (14.7)	14 (11.4)	0.25
6 weeks postpartum (*n* = 80)				
Need for healthcare professional (n, %)	4 (5.0)	1 (2.3)	3 (8.1)	0.33

*N* number of participants, *SD* standard deviation, *NICU* neonatal intensive care unit

**Table 4:** Association between adhesions and maternal and neonatal outcomes

Maternal outcomes	No adhesions *n* = 207	Adhesions *n* = 124	
		**Unadjusted β (95% CI)**	**Adjusted β (95% CI)** ^**a**^
Maternal outcomes			
Perioperative outcomes (*n* = 335)			
Operation time (minutes)	Ref	9.81 (6.60-13.01)	9.58 (6.39-12.77)
Infant delivery time (minutes)	Ref	2.36 (1.32-3.41)	2.35 (1.29-3.39)
Perioperative blood loss (ml)	Ref	26.30 (−9.08-61.68)	22.69 (−12.34-57.73)
Postoperative outcomes at discharge (*n* = 335)			
Length of hospital stay (days)	Ref	−0.15 (−0.37-0.07)	−0.15 (−0.37-0.07)
6 weeks postpartum (*n* = 80)		**OR (95% CI)**	**aOR (95% CI)**
Fever	Ref	1.17 (0.16-8.75)	1.68 (0.15-9.00)
Wound infection	Ref	1.39 (0.32-5.88)	1.38 (0.32-5.96)
Daily use of painkiller	Ref	2.40 (0.21-27.59)	2.30 (0.20-26.49)
Able to resume daily activities	Ref	1.24 (0.48-3.23)	1.19 (0.44-3.16)
Abnormal bleeding	Ref	1.18 (0.32-4.47)	1.16 (0.30-4.44)
Need for healthcare professional	Ref	0.53 (0.15-1.93)	0.59 (0.16-2.23)
Neonatal outcomes Perioperative (*n* = 335)			
Apgar score at 5 min <7	Ref	1.16 (0.45-2.98)	1.18 (0.45-3.08)
NICU admission	Ref	0.47 (0.16-1.40)	0.46 (0.15-1.38)
6 weeks postpartum (*n* = 80)			
Need for healthcare professional	Ref	3.71 (0.36-37.26)	4.55 (0.37-55.05)

^a^Adjusted for maternal weight at 20 weeks gestation and presence of uterine fibroids. NICU = neonatal intensive care unit

## Discussion

This study observed that the majority of women with a history of CS or abdominal surgery had adhesions, and this affected operation time, infant delivery time, and perioperative blood loss (for severe adhesions), but not other outcomes. The prevalence and severity of adhesions increased with each additional repeat CS.

These findings concur with earlier studies that found adhesions to be a frequent complication after CS [9, 12, 13, 17–22]. The prevalence of adhesions in this population in Ghana is higher than reported in previous studies [12]. This could be a reflection of a higher prevalence of complex cases at a major referral teaching hospital in this region, which may also explain the high rate of CS conducted at KBTH (43% of deliveries). Alternatively, the prevalence of post operative adhesions in low and middle-income countries may be higher as the performance of a surgical procedure is subject to adequately trained medical personnel, available infrastructure and access to resources [18, 23–26]. Therefore, and reinforced by the increase in CS rates, there is an urgent need for data of post operative complications such as adhesions from low and middle income countries. Such prospective studies should include detailed information of procedures and complications during the first CS.

We observed primarily perioperative consequences of adhesions. These factors such as total operation time and infant delivery time can have clinical consequences at time of emergency procedures due to fetal or maternal distress. Further, even in non-emergency situations, increased operation time requires prolonged anesthesia [27] and exposure to infection risk [28]. The increase in blood loss with (severe) adhesions is relevant in light of the persistent high prevalence of anemia in pregnant women in low- and middle income country settings [29].

### Strengths and limitations

Strengths of this study include the prospective design in which women were recruited before CS. The nested sub-study included women at 6 weeks postpartum and this allowed to not only assess perioperative outcomes, but explore important maternal and neonatal short and mid-term outcomes as postpartum blood loss, sepsis, wound infection, and postpartum need for a healthcare professional as well. Another strength of this study is the use of a standardized adhesion classification system, making it possible to quantify the presence and severity of adhesions in a systematic manner.

A number of limitations need to be considered in the interpretation of the data. First, because the research staff could not be present 24 h, 7 days a week at the facility, 20% of the eligible study base was included. This could have introduced selection bias, with more elective cases included potentially resulting in in higher operation and infant delivery times, but an underestimation of poor maternal and neonatal outcomes. Another limitation of the design of our study is the size of the 6 weeks postpartum follow-up group (*n* = 80), which could contribute to a lack of power to observe a difference in outcomes. Women followed up at 6 weeks were different in education level, more often had a neonatal indication for CS, and were more likely to have an elective CS (data not shown), possibly limiting generalizability of these findings. In busy obstetric settings such as KBTH including additional research visits create a lot of extra work pressure for the staff in limited available workspace. We expected the follow up of women 6 weeks post-partum to involve quite some additional efforts for the research team and therefore we planned to limit the follow up to the first 5 weeks of the study. However we could have ensured active follow up throughout the whole study, as there was generally a high follow up rate at six-week postpartum. For future studies we will include postpartum follow up for all women, or women could be followed by phone or home visits. A third limitation was the occurrence of missing data records for 79 participants initially enrolled. Because we assume this occurred randomly, and this was <20% of the population, we do not expect this to strongly affected the study outcomes. Fourth, as this study was powered for the incidence of adhesions, and not other complications, sample size limitations could have contributed to the lack of significant findings for other complications. Finally, details on previous CS indication or surgical techniques were often lacking - reflective of the setting where this study took place: a major referral hospital in a low resources setting that received patients from most part of the country without detailed description of previous surgery history available. Future research could address some limitations in prospective cohorts starting at the first CS that follow pregnant women up until future pregnancies. Future studies could also investigate factors affecting the occurrence of adhesions including surgical techniques, and strategies to prevent the development of adhesions and other complications - as explored in the CORONIS studies [28–31].

### Implication for clinical practice

Unfortunately, there is no gold standard to avoid adhesions or detect adhesions prior to surgery [16]. Surgical training, careful removal of debris and blood, and reducing the risk of infections will all contributes to reduced incidence [16, 32]. The use of barriers, peritoneal suturing, as well as various other closure techniques have not resulted in satisfactory reduction of adhesions occurrence [33, 34]. Therefore, pregnant women need to be carefully counseled about their options and associated implications, especially before and during the first pregnancy after their first CS to consider a vaginal birth after cesarean section (VBAC) or when requesting a CS for non-medical indication.

## Conclusion

The majority of women with a history of CS or abdominal surgery had adhesions and this affected operation time, infant delivery time, and perioperative blood loss (for severe adhesions), but not other outcomes. With each additional repeat CS, prevalence and severity of adhesions increased. With the global increase in cesarean sections in general and particularly repeat cesareans, short- and long-term consequences will need to be considered and discussed with pregnant women during preoperative counseling. Notwithstanding the need for all people to have access to high quality safe surgical care [35], there is the concurrent responsibility to avoid unnecessary first cesarean sections [8].

## Additional files

Additional file 1: Table S1. Presence and severity of adhesions by number of previous cesarean section. (DOCX 10 kb)

Additional file 2: Table S2. Baseline characteristics of participating women by adhesion group. (DOCX 14 kb)

Additional file 3: Table S3. Associations of adhesion groups by score and classification. (DOCX 15 kb)

## Abbreviations

ANOVA: Analysis of variance
CS: Cesarean section
KBTH: Korle Bu Teaching Hospital
NICU: Neonatal intensive care unit
ORs: Odds ratios
STROBE: STrengthening the Reporting of OBservational studies in Epidemiology
VBAC: Vaginal births after cesarean
WHO: World Health Organization
