# Supplementary material for: Prevalence of adhesions and associated postoperative complications after cesarean section in Ghana: a prospective cohort study
Source: Reprod Health. 2017 Nov 2;14:143. doi: 10.1186/s12978-017-0388-0 (PMC5667441; doi:10.1186/s12978-017-0388-0)
Supplement: Supplementary file 1 — Presence and severity of adhesions by number of previous cesarean section. (DOCX 10 kb) [file 12978_2017_388_MOESM1_ESM.docx]

Additional file 1: Table S1: Presence and severity of adhesions by number of previous cesarean section.

| Adhesion category | No previous CS (n=107) | 1 previous CS (n=150) | >1 previous CS (n=75) |
| --- | --- | --- | --- |
| No adhesions | 104 (97.2) | 73 (48.7) | 30 (38.5) |
| Mild adhesions | 1 (0.9) | 41 (27.3) | 25 (32.0) |
| Severe adhesions | 2 (1.9) | 36 (24.0) | 23 (29.5) |
