# Supplementary material for: Prevalence of adhesions and associated postoperative complications after cesarean section in Ghana: a prospective cohort study
Source: Reprod Health. 2017 Nov 2;14:143. doi: 10.1186/s12978-017-0388-0 (PMC5667441; doi:10.1186/s12978-017-0388-0)
Supplement: Supplementary file 2 — Baseline characteristics of participating women by adhesion group. (DOCX 14 kb) [file 12978_2017_388_MOESM2_ESM.docx]

Additional file 2: Table S2: Baseline characteristics of participating women by adhesion group

|  | No adhesions | Mild adhesions | Severe adhesions | P-value |
| --- | --- | --- | --- | --- |
|  | n=207 | n=67 | n=61 |  |
| *Socio-demographic characteristics* |  |  |  |  |
| Age(years in mean, SD) | 30.6 ± 5.2 | 32.5 ± 4.6 | 32.2 ± 3.9 | <0.01 |
| Current living in Greater Accra region (n, %) | 191 (92.7) | 55 (84.6) | 52 (86.7) | <0.01 |
| Married, engaged or living together (n, %) | 174 (87.0) | 60 (89.6) | 52 (86.3) | 0.76 |
| Education level (n, %) |  |  |  | 0.73 |
| No education | 17 (8.4) | 4 (6.1) | 7 (11.5) |  |
| Primary school | 59 (29.1) | 21 (31.8) | 14 (23.0) |  |
| Secondary school | 69 (34.0) | 25 (37.9) | 19 (31.2) |  |
| Tertiary school | 58 (28.6) | 16 (24.2) | 21 (34.4) |  |
| *Obstetric and medical history* |  |  |  |  |
| Parity (n, %) |  |  |  | <0.01 |
| 0 | 63 (30.4) | 0 (0.00) | 2 (3.3) |  |
| 1-4 | 137 (66.2) | 65 (97.0) | 57(95.0) |  |
| 5-9 | 7 (3.4) | 2 (3.0) | 1 (1.7) |  |
| Number of previous CS (n, %) |  |  |  | <0.01 |
| 0 | 104 (50.2) | 1 (1.5) | 2 (3.3) |  |
| 1 | 73 (35.3) | 41 (61.2) | 36 (59.0) |  |
| 2 | 27 (13.0) | 22 (32.8) | 12 (19.7) |  |
| 3 | 3 (1.5) | 3 (4.5) | 11 (18.0) |  |
| Indication for previous CS (n, %) |  |  |  | 0.21 |
| Maternal | 21 (21.4) | 22 (37.9) | 18 (32.7) |  |
| Maternal-neonatal | 40 (40.8) | 21 (36.2) | 21 (38.1) |  |
| Neonatal | 37 (37.8) | 15 (25.9) | 16 (29.1) |  |
| Previous surgery (n, %) | 0 (0.0) | 7 (10.6) | 10 (16.4) | <0.01 |
| Uterine fibroids | 6 (2.9) | 2 (3.0) | 4 (6.6) | 0.37 |
| Mid-pregnancy weight in kg (mean, SD) | 73.8 (15.0) | 72.9 (12.8) | 77.6 (13.6) | 0.19 |
|  |  |  |  |  |
| *Current delivery* |  |  |  |  |
| Gestational age at delivery in weeks (n, %) |  |  |  | 0.41 |
| ≤37 | 51 (27.1) | 16 (25.4) | 11 (19.6) |  |
| 38 – 41 | 131 (69.7) | 44 (69.8) | 45 (80.4) |  |
| ≥42 | 6 (3.2) | 3 (4.8) | 0 (0.0) |  |
| Type of current CS |  |  |  | 0.05 |
| Elective | 68 (32.9) | 32 (47.8) | 27 (44.3) |  |
| Emergency | 139 (67.2) | 35 (52.2) | 34 (55.7) |  |
| Indication for current CS (n, %) |  |  |  | <0.01 |
| Maternal | 84 (41.8) | 51 (77.3) | 38 (63.3) |  |
| Maternal-neonatal | 57 (28.4) | 12 (18.2) | 14 (23.3) |  |
| Neonatal | 60 (29.6) | 3 (4.6) | 8 (13.3) |  |

CS= cesarean section, n= number, SD= standard deviation
