# Supplementary material for: Prevalence of adhesions and associated postoperative complications after cesarean section in Ghana: a prospective cohort study
Source: Reprod Health. 2017 Nov 2;14:143. doi: 10.1186/s12978-017-0388-0 (PMC5667441; doi:10.1186/s12978-017-0388-0)
Supplement: Supplementary file 3 — Associations of adhesion groups by score and classification. (DOCX 15 kb) [file 12978_2017_388_MOESM3_ESM.docx]

Additional file 3: Table S3: Associations of adhesion groups by score and classification

| **Maternal outcomes** | **Adhesion score (continuous)** | | **Adhesions (mild, severe)** | |
| --- | --- | --- | --- | --- |
| **Perioperative outcomes** | **Unadjusted β (95% CI)** | **Adjusted β (95% CI)*** | **Unadjusted β (95% CI)** | **Adjusted β (95% CI)*** |
| Operation time (minutes) | 0.48 (0.36-0.61) | 0.47 (0.35-0.60) | 5.91 (1.97-9.84)  14.18 (10.08-18.29) | 5.88 (1.97-9.80)  13.80 (9.67-17.93) |
| Infant delivery time (minutes) | 0.11 (0.07-0.15) | 0.11 (0.07-0.15) | 1.73 (0.43-3.03)  3.07 (1.72-4.41) | 1.73 (0.44-3.04)  3.02 (1.66-4.38^)^ |
| Perioperative blood loss (ml) | 2.39 (1.00-3.77) | 2.20 (0.92-3.57) | -6.98 (-50.68-36.71)  63.53 (17.93-109.14) | -7.85 (-51.03-35.33)  57.55 (12.09-103.01) |
| **Postoperative outcomes at discharge** |  |  |  |  |
| Length of hospital stay (days) | -0.0 (-0.01 - 0.01) | -0.00 (-0.01 - 0.01) | -0.22 (-0.50 - 0.05)  -0.08 (-0.36-0.21) | -0.22 (-0.50-0.05)  -0.06 (-0.35-0.22) |
| **6 weeks postpartum (n=80)** | **OR (95% CI)** | **aOR (95% CI)** | **OR (95% CI)** | **aOR (95% CI)** |
| Fever or infection | 1.00 (0.92-1.08) | 1.00 (0.92-1.08) | 0.89 (0.08 - 10.37)  1.71 (0.14-20.51) | 0.90 (0.08 - 10.80)  1.69 (0.13-121.83) |
| Daily use of painkiller | 1.02 (0.95-1.09) | 1.02 (0.95-1.09) | 1.83 (0.11-30.58)  3.5 (0.30-60.21) | 1.76 (0.10-29.90)  3.25 (0.19-56.76) |
| Able to resume daily activities | 1.02 (0.98-1.06) | 1.02 (0.98-1.06) | 1.06 (0.35-3.21)  1.61 (0.44-5.93) | 0.97 (0.32-3.00)  1.72 (0.45-6.59) |
| Abnormal bleeding | 1.01 (0.97-1.06) | 1.01 (0.97-1.06) | 0.69 (0.12-3.87)  2.28 (0.46-11.20) | 0.69 (0.12-3.94)  2.10 (0.42-10.5) |
| Need for healthcare professional | 1,00 (0.95-1.05) | 1.00 (0.95-1.05) | 0.62 (0.15-2.62)  0.36 (0.04-3.22) | 0.70 (0.16-3.02)  0.41 (0.05-3.73) |
| **Neonatal outcomes (n=334)** |  |  |  |  |
| Apgar score at 5 min. ≤7 | 1.03 (0.97-1.09) | 1.03 (0.97-1.09) | 0.83 (0.28 - 2.42)  1.97 (0.43-9.01) | 0.93 (0.28-2.42)  2.11 (0.45-9.87) |
| NICU admission | 0.47 (0.16-1.40) | 0.94 (0.87-1.02) | 0.65 (0.20-2.15)  0.22 (0.03-1.81) | 0.66 (0.20-2.20)  0.21 (0.03-1.71) |
| **6 weeks postpartum (n=80)** |  |  |  |  |
| Need for healthcare professional | 3.71 (0.37-37.26) | 1.00 (0.92-1.09) | 3.81 (0.32-44.50)  3.50 (0.20-60.21) | 5.04 (0.35-71.87)  3.86 (0.19-77.17) |
| *adjusted for maternal weight at 20 weeks gestation and presence of uterine fibroids | | | | |
